# Optimization and Analysis of Large-Aperture Ultrathin Mirror Based on Multiphysics Coupling

**DOI:** 10.3390/s26134188

**Published:** 2026-07-02

**Authors:** Yuzhe Wang, Zhonghuai Wu

**Affiliations:** 1 School of Interdisciplinary Science, Beijing Institute of Technology, Beijing 100081, China; 2 Yangtze Delta Region Academy of Beijing Institute of Technology, Jiaxing 314019, China

**Keywords:** multiphysics coupling, large-aperture ultrathin mirror, lightweighting, optimization method, simulation analysis

## Abstract

**Highlights:**

**What are the main findings?**

- A thermo-mechanical physical field coupling mechanism was established, and a topology optimization method based on multi-physics field coupling was proposed.
- By combining the advantages of topology optimization and parameter optimization, the optimized mirror exhibits better thermodynamic performance and a higher mass reduction ratio.

**What are the implications of the main findings?**

- This optimization method considers the influence of thermo-mechanical physical fields on large-aperture ultrathin mirrors, which helps to improve the mirror’s overall performance indicators such as surface accuracy, environmental adaptability, and stiffness characteristics.
- This optimization method can provide new ideas for the optimized design of lightweight mirrors with strong environmental adaptability.

**Abstract:**

As a key component of space telescopes, the rational structure of the mirror is a crucial factor affecting the telescope’s environmental adaptability and imaging performance. To address the technical challenges of simultaneously achieving lightweight, environmental adaptability, and surface accuracy in large-aperture ultrathin mirrors, this paper proposes a mirror optimization method based on multiphysics coupling. Based on the finite element method and thermoelasticity theory, the interaction relationship between the temperature physical field and the force physical field was established. The P-norm was used to solve the problems of non-smoothness and inability to solve the sensitivity of the max(·) function. An optimized model for the mirror was determined using a combination of topology optimization and parameter optimization. Compared to a solid mirror, the optimized mirror achieved a mass reduction rate of 82.04%. Under temperature and gravity conditions, the surface accuracy of the optimized mirror met the requirements. In terms of response dynamics, the optimized mirror performs better, with a maximum response amplification factor of 4.39, below the threshold of 4.5 required to maintain structural stability, which is crucial for maintaining the structural integrity of the mirror. This method will provide a feasible approach for the optimized design of lightweight mirrors with strong environmental adaptability.

## 1. Introduction

As an important technology for target detection, space telescopes utilize large-aperture ultrathin mirrors, which not only improve the telescope’s detection accuracy but also enhance its detection performance [1,2,3,4,5,6]. As the size of the mirrors continues to increase, its mass will increase in a power function manner. At the same time, there are significant differences in the physical fields of gravity and temperature between the Earth’s surface and space [7,8]. A well-designed lightweight mirror structure can reduce the mirror’s mass while improving the mirror’s surface accuracy under temperature and gravitational loads, thereby enhancing the mirror’s adaptability to fluctuations in multiphysics environments. However, it should be noted that whether the mirror’s surface accuracy can be improved during material removal depends on the rate of change of the stiffness-to-mass ratio [9,10,11]. The key challenge in the development of space telescopes lies in achieving a high mass reduction ratio while ensuring that large-aperture ultrathin mirrors possess good surface accuracy, stiffness characteristics, and environmental adaptability.

Many scholars have conducted research on the problem of mirror optimization and have made significant progress. Zeng et al. [12] proposed a TPMS structure optimization design method based on a homogenization performance model. This method can accurately describe the performance of TPMS structures and obtain high-performance structures with minimal computational cost. The optimized mirror structure has both high mass reduction rate and low shape error. Yan et al. [13] combined additive manufacturing and topology optimization methods, used theoretical calculations to determine the mirror support scheme and thickness parameters, and used the lattice filling method and an equivalent analysis method to realize the passive integration and heatless design of the mirror assembly. Shen et al. [14] proposed an overall double-sided mirror topology optimization method with controlled surface error. The root mean square error of the surface shape of the primary mirror under axial gravity is used as the objective function, and the total mass is used as the constraint condition. Compared with the traditional optimization method, this method improves the surface shape accuracy of the optimized mirror. Jiang et al. [15] proposed a topology optimization algorithm that satisfies the material volume minimization and buckling constraint conditions under design load. They used the SIMP method combined with a pressure boundary search scheme based on the DRLSE model to handle design-irrelevant loads. By introducing the K-S aggregation function, multiple constraints were simplified into a single constraint, which replaced the original buckling constraint conditions. Sun et al. [16] proposed an intelligent multi-objective parameterization method for mirror optimization. By constructing a complex objective function with the mirror shape error and total mass as optimization objectives, the mirror shape error problem can be solved, avoiding the tedious steps of manually adjusting the model. Liu et al. [17] improved the initial structure of the mirror and optimized the initial structural parameters of the improved primary mirror and flexible hinge based on the parameter optimization algorithm of the compromise programming mathematical method. Dong et al. [18] proposed a multi-objective optimization method based on dual-parameter coupled performance analysis. Through artificial intervention and performance law, the optimal solution of the multi-objective function of the mirror was obtained. Tan et al. [19] proposed a multi-objective optimization method for mirrors based on the dynamic constraint shamode algorithm, which solved the multi-objective optimization problem of simultaneously minimizing mass and flexibility, controlling surface error and first-order modal frequency under strict constraints. Koppen et al. [20] proposed a new technique that combines structural-thermal-optical performance analysis with topology optimization, taking into account both component-level and system-level constraints. The proposed method can significantly reduce the system spot size error under the same constraints. Shojaee et al. [21] proposed a topology optimization algorithm based on the level set method, which combines the topological derivative with the nonlinear level set equation, solving the problems of premature convergence of the algorithm and the high dependence of the optimal topology on the initial design. Unlike the explicit scheme commonly used in the traditional level set method, this method uses a semi-implicit additive operator splitting scheme to solve the level set equation, and implements a truncation strategy to limit the range of maximum and minimum values in the design domain. In response to the design requirements of lightweight and high surface shape accuracy of space mirrors, Fan et al. [22] proposed a design and manufacturing method that combines topology optimization and additive manufacturing technology. With the minimization of structural compliance as the optimization objective and mass fraction as the design constraint, the topology optimization design of the mirror was realized, and the optimized structure was manufactured by additive manufacturing. Dong et al. [23] used the MOAT and Sobol methods to determine the key parameters affecting the design, took the high-sensitivity component as the optimization domain, and realized the optimized design of the structure based on the multi-objective SIMP topology optimization method. They also conducted optical-thermal coupling analysis on the optical system. Liu et al. [24] proposed a topology optimization design method for active deformable mirrors based on discrete orthogonal Zernike polynomials. The method uses discrete orthogonal Zernike polynomials to characterize the wave aberration of the active deformable mirror, and combines optical and structural deformation to establish an optical-mechanical coupling topology optimization model and derive the sensitivity of the mathematical model.

Many scholars have conducted research on how to maintain the surface accuracy of the mirror, improve the optimization efficiency, and increase the structural stiffness during the optimization process. Some scholars have considered the influence of gravity or temperature as a single physical field during the optimization process, while others have also conducted thermo-optical coupling analysis on the optimized mirror. However, there are few reports on topology optimization studies that take large-aperture ultrathin mirrors as the research target and consider the coupling effect of gravity and thermal physical fields. In-depth research on the relationship between coupled physical fields and mirror optimization is not only of great scientific significance, but can also solve the technical difficulties of high-quality imaging in practical engineering.

To address the impact of multiphysics coupling on the lightweighting process of mirrors, this paper proposes a mirror optimization method based on multiphysics coupling. This method explores the coupling effect of temperature and gravity, clarifies the influence of coupled physical fields on mirror optimization, and solves the key problem of simultaneously achieving mass reduction rate, structural stiffness, and surface accuracy. In the optimization process, in view of the optimization limitations and instability caused by the non-smoothness of the displacement constraint function, this paper adopts the P-norm to make a smooth approximation of the max(·) function, which solves the problems of non-smoothness of the original function and inability to solve the sensitivity, and strictly maintains its own convexity. In addition, the key geometric parameters of the topology-optimized mirror were further optimized by traversing all parameters to determine the optimal parameter set. This paper presents a comprehensive simulation analysis using the final optimized mirror as the research object, and sets up two control groups. The analysis results demonstrate the correctness and reliability of the proposed method.

The structure of this paper is as follows: Section 2 details the theoretical derivation of the optimization method, including the principle of thermo-mechanical physical field coupling, the numerical model of topology optimization, the equation for solving sensitivity, and the method for fitting the surface shape accuracy of the mirror; Section 3 presents the optimized design of the mirror; Section 4 simulates and analyzes the optimized mirror, mainly involving thermo-mechanical and mechanical analysis; Section 5 discusses the results; and Section 6 summarizes the conclusions of this study.

## 2. Theoretical Research

### 2.1. General Formulation of Thermo-Mechanical Physical Field Coupling

**Figure 1:**
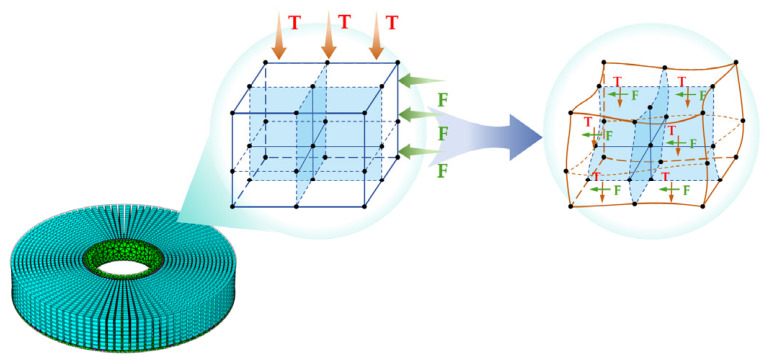
Thermo-mechanical physical field coupling effect on a three-dimensional finite element model.

Temperature and gravitational forces directly affect the mirror material, causing structural stress and deformation, which severely impacts the surface accuracy of lightweight mirrors. For the thermo-elastic optimization problem, Figure 1 shows the basic thermo-mechanical coupling model of the three-dimensional continuum structure, which includes the design domain, non-design domain, geometric boundary conditions, and load conditions.

Based on the finite element method and thermo-elastic theory, the steady-state equilibrium equation of the structure under the combined action of thermal and mechanical loads can be expressed as follows:

(1) $$K(\rho )U(\rho )=F_{m}+F_{t}(\rho )$$

where K(ρ) is the global stiffness matrix, U(ρ) is the global displacement matrix, $F_{m}$ is the external force load, $F_{t}(\rho )$ is the equivalent thermal load, ρ is the element relative density variable vector, and $\rho ={\left(\rho_{1},\rho_{2},\cdots ,\rho_{e},\cdots \rho_{n}\right)}^{T}$.

In Equation (1), the global stiffness matrix K(ρ) can be expressed by the element stiffness matrix as follows:

(2) $$K(\rho )={∭}_{\Omega_{e}}B_{e}^{T}D_{e}(\rho_{e})B_{e}h_{e}d\Omega$$

where $B_{e}$ is the element strain matrix, $D_{e}(\rho_{e})$ is the element elasticity matrix, $h_{e}$ is the element thickness, and Ω is the design domain.

The element elasticity matrix $D_{e}(\rho_{e})$ can be expressed as

(3) $$D_{e}(\rho_{e})=E(\rho_{e}){\overset{-}{D}}_{e,c}$$

where ${\overset{-}{D}}_{e,c}$ is the constant term of the element elasticity matrix, which is related to the material’s Poisson’s ratio, and $E(\rho_{e})$ is the element elastic modulus, which depends on the relative density variable.

Substituting Formula (3) into Formula (2) yields the following result:

(4) $$K(\rho )=E(\rho_{e}){∭}_{\Omega_{e}}B_{e}^{T}{\overset{-}{D}}_{e,c}B_{e}h_{e}d\Omega$$

The equivalent thermal load $F_{t}(\rho )$ is a load dependent on design variables and can be assembled from the thermal load vectors of the element nodes, expressed as follows:

(5) $$F_{t}(\rho )={∭}_{\Omega_{e}}B_{e}^{T}D_{e}(\rho_{e})\varepsilon_{e}^{t}(\rho_{e})h_{e}d\Omega$$

where $\varepsilon_{e}^{t}(\rho_{e})$ is the element thermal strain tensor, and its calculation formula is:

(6) $$\varepsilon_{e}^{t}(\rho_{e})=\alpha (\rho_{e})∆t_{e}\phi^{T}$$

In the above formula, $\alpha (\rho_{e})$ is the thermal expansion coefficient related to the relative density of the element, $∆t_{e}$ is the temperature change of the element, which can be approximated by the difference between the average temperature of each node of the element and the reference temperature, and ϕ is a constant vector [1, 1, 1, 0, 0, 0].

Substituting Equations (3) and (6) into Equation (5), the equivalent thermal load $F_{t}(\rho )$ caused by temperature change in a three-dimensional continuous structure can be expressed as:

(7) $$F_{t}(\rho )=E(\rho_{e})\alpha (\rho_{e}){∭}_{\Omega_{e}}B_{e}^{T}{\overset{-}{D}}_{e,c}∆t_{e}\phi^{T}h_{e}d\Omega$$

Through the above derivation, the coupling relationship between thermal and mechanical physical fields was established, which is also an important basic condition for establishing a connection between coupled physical fields and topology optimization.

### 2.2. Mathematical Model of Topology Optimization

Unlike traditional optimization methods, topology optimization is a mathematical method that seeks the optimal material layout and the best force transmission path within the structural design domain. It does not require consideration of the initial structural configuration and can obtain a completely new topological form of the structure based on constraints.

For large-aperture ultrathin mirrors, the mechanical properties and surface accuracy are affected by the removal of material. Therefore, the stiffness of the mirror is a primary indicator to consider during topology optimization. This paper uses the minimization of structural compliance as the objective function and maximizes the mirror stiffness by constraining the remaining material volume and nodal displacements. The structural compliance is expressed as:

(8) $$C=\frac{1}{2}F^{T}U$$

In the formula, F is the equivalent load, i.e., $F=F_{m}+F_{t}(\rho )$.

For the material interpolation model, this paper used Solid Isotropic Material with Penalization (SIMP) to establish the functional relationship between the element elastic modulus and the relative density variable of the material [25,26,27].

(9) $$E(\rho_{e})=\rho_{e}^{p}E_{0}$$

In the formula, $E_{0}$ is the elastic modulus of the solid material, and p is the penalty factor. The value is 3, which is used to “penalize” the relative density variable to retain or remove material at the corresponding location of the element. To avoid singularity of the stiffness matrix in numerical calculations, the relative density variable should have a minimum positive lower bound constraint value. In addition, choosing a reasonable penalty factor value can avoid getting trapped in local optima and generating too many gray-scale elements.

A mathematical model for topology optimization of a mirror based on thermophysical field coupling is proposed. The objective function is to minimize the structural compliance, with volume constraints and nodal displacement constraints as conditions. The numerical optimization model is as follows:

(10) $$\begin{array}{ll} \min & C=\frac{1}{2}F^{T}U\\ & \;K(\rho )U(\rho )=F_{m}+F_{t}(\rho )\\ \begin{array}{l} s.t. \end{array} & \begin{array}{l} K_{t}(\rho )T(\rho )=Q\\ \sum_{e=1}^{N_{e}}\rho_{e}v_{e}\leq \overset{-}{V}\times V_{0}\\ \begin{array}{l} \max\left(\left|u_{i}\right|\right)\leq \overset{-}{u}\;(i=1,2,\cdots ,N_{p})\\ \;0<\rho_{\min}\leq \rho_{e}\leq 1\;(e=1,2,\cdots ,N_{e}) \end{array} \end{array} \end{array}$$

In the formula, $K_{t}(\rho )$ is the global heat conduction matrix, T(ρ) is the global temperature matrix, Q is the heat flux, $\overset{-}{V}$ is the volume fraction ($\overset{-}{V}$ = 0.3 in this paper), $V_{0}$ is the volume of the solid structure, $\max\left(\left|u_{i}\right|\right)$ is the maximum displacement of the node, $\overset{-}{u}$ is the upper limit constraint value of the node displacement, $N_{p}$ is the number of global nodes, $\rho_{\min}$ is the lower limit constraint value of the relative density variable (taken as 0.001), and $N_{e}$ is the number of global elements.

### 2.3. Sensitivity Analysis

This paper uses the Method of Moving Asymptotes (MMA) [28] to solve the topology optimization problem with respect to Formula (10), and sensitivity calculation is the core step in the iterative solution process.

The sensitivity of compliance to the relative density variable is expressed as:

(11) $$\frac{\partial C}{\partial \rho_{e}}=U^{T}\frac{\partial F_{t}}{\partial \rho_{e}}-\frac{1}{2}U^{T}\frac{\partial K}{\partial \rho_{e}}U$$

Volume constraint sensitivity is expressed as

(12) $$\sum_{e=1}^{N_{e}}\frac{{\partial \rho }_{e}v_{e}}{\partial \rho_{e}}=v_{e}$$

The displacement constraints are simplified and expressed as follows:

(13) $$g(\rho )=\max(\left|u_{i}\right|)-\overset{-}{u}$$

max(·) is a piecewise continuous convex function. At the critical point where inputs are equal, the sensitivity cannot be solved, and its non-smoothness leads to convergence oscillations and low solution efficiency. Therefore, this paper uses the p-norm to smoothly approximate max(·), achieving gradient solution while improving computational accuracy and convergence speed. Equation (13) can be rewritten as:

(14) $$g(\rho )={\left(\sum_{i}^{N_{p}}{\left|u_{i}\right|}^{\mathrm{pn}}\right)}^{\frac{1}{\mathrm{pn}}}-\overset{-}{u}$$

where $u_{i}$ represents the nodal displacement component.

The displacement constraint sensitivity can be expressed using the adjoint method and the chain rule.

(15) $$\frac{\partial g}{\partial \rho_{e}}=-\frac{1}{{\left\|U\right\|}_{\mathrm{pn}}^{\mathrm{pn}-1}}\cdot \lambda^{T}\cdot \frac{\partial K}{\partial \rho_{e}}\cdot U$$

where ${\left\|U\right\|}_{\mathrm{pn}}$ is the p-norm approximation of $\max(\left|u_{i}\right|)$. When pn approaches +∞, it can be approximated that ${\left\|U\right\|}_{\mathrm{pn}}=\max(\left|u_{i}\right|)$. λ is the adjoint vector, satisfying Kλ = M, where **M** is the auxiliary vector.

### 2.4. Methods for Fitting Surface Shape Accuracy

The effects of temperature and force loads on the surface accuracy of a reflective mirror are typically represented by the root mean square (RMS) of the surface deviation. Using the finite element method, the mirror surface is discretized and uniformly sampled, the RMS of the surface deviation is expressed as:

(16) $$\mathrm{RMS}=\sqrt{\frac{1}{N_{a}}\sum_{i=1}^{\mathrm{Na}}∆h_{i}^{2}}$$

where $N_{a}$ is the number of sampling points; the more sampling points there are, the more accurate the surface deviation calculation will be. $∆h_{i}$ is the local normal displacement deviation controlled by the sampling points.

## 3. Optimization of the Mirror

From a functional perspective, a mirror can be divided into two parts: the mirror surface and the mirror body. The main function of the mirror surface is to reflect and adjust incident light, while the main function of the mirror body is to maintain the mirror surface’s accuracy and rigidity. Although retaining more mirror body material can, to some extent, give the mirror good mechanical properties, excessive mass can also lead to significant problems such as decreased mirror surface accuracy and reduced heat dissipation. The core research content of this paper is to find a globally optimal solution under constraints while simultaneously reducing mirror body material and maintaining the mirror’s surface accuracy.

### 3.1. Discretized Representation of the Mirror

This paper takes a large-size mirror as the research object. To ensure that the reflector has good structural rigidity, temperature characteristics, and machinability [29], SiC is selected as the material. SiC material possesses excellent thermal properties, exhibiting high thermal conductivity, low coefficient of thermal expansion, and good stability at high temperatures. Simultaneously, it also possesses outstanding mechanical properties, with high stiffness and low density. Whether for lightweighting mirrors or adaptability to extreme environments, SiC material offers significant advantages. Table 1 provides detailed parameters for the mirror. The mirror’s position is fixed via a central mandrel, a support method characterized by its simple structure and ease of fabrication.

**Figure 2:**
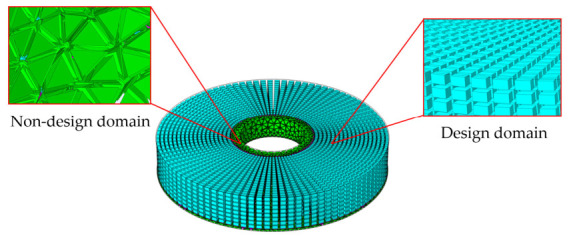
Finite element model of a mirror.

According to the optimization requirements, the mirror surface is the non-design domain, and the mirror body is the design domain. To improve the calculation accuracy, this paper uses free tetrahedral elements to discretize the non-design domain (16,733 meshes) and hexahedral elements to discretize the design domain (10,080 meshes). The optimization convergence residual threshold is 0.001, the solver type is Multifrontal Massively Parallel Solver, and the maximum number of iterations is 100. Figure 2 shows the finite element model of the mirror.

### 3.2. Optimized Design of the Mirror

**Figure 3:**
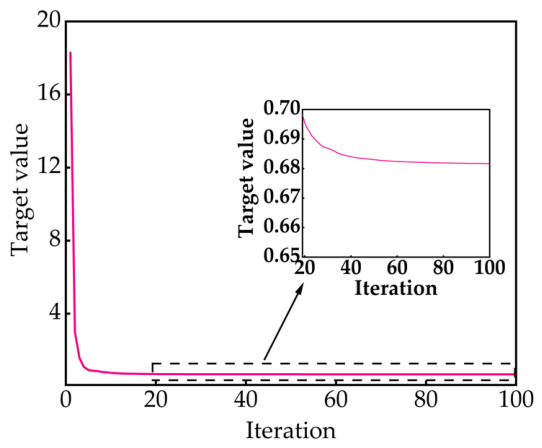
Iteration curve of objective function.

The construction and solution method of the mathematical model for topology optimization of the mirror under the coupling effect of thermophysical fields can be referred to the formula derivation in Section 2. Since the mechanical load in this paper is gravity, when mechanical load is involved in Formulas (1)–(15), it is only necessary to associate it with the relative density variable. In the topology optimization process, the central axis of the mirror is fully constrained and fixed, the global temperature condition is set to 21.5 °C, the gravity load acts on the entire domain, and the two are coupled in the form of small deformation. As can be seen from Figure 3, the objective function shows a monotonically converging trend with the continuous iteration of the optimization variables. After 40 iterations, the objective function basically converges.

**Figure 4:**
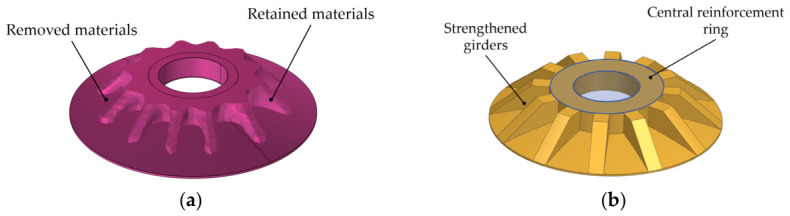
Distribution of mirror material: (**a**) Topology optimization model; (**b**) Intermediate model.

The optimized structure of the mirror is shown in Figure 4a. Most of the material was removed, leaving only twelve trapezoidal reinforcing ribs. These ribs are circumferentially symmetrical, with material stacked radially around the central core. At the 1/2 radial position of the design domain, the material stacking pattern changes; it is no longer a uniform stacking shape, but rather the stacked material gradually decreases as the radial distance increases. The ends of the reinforcing ribs are notched. The reinforcing ribs are not completely independent but are constructed as a unit through a central reinforcing ring. To improve the manufacturability of the mirror, further optimization was performed based on the optimized topology (referred to as the intermediate model), as shown in Figure 4b.

**Figure 5:**
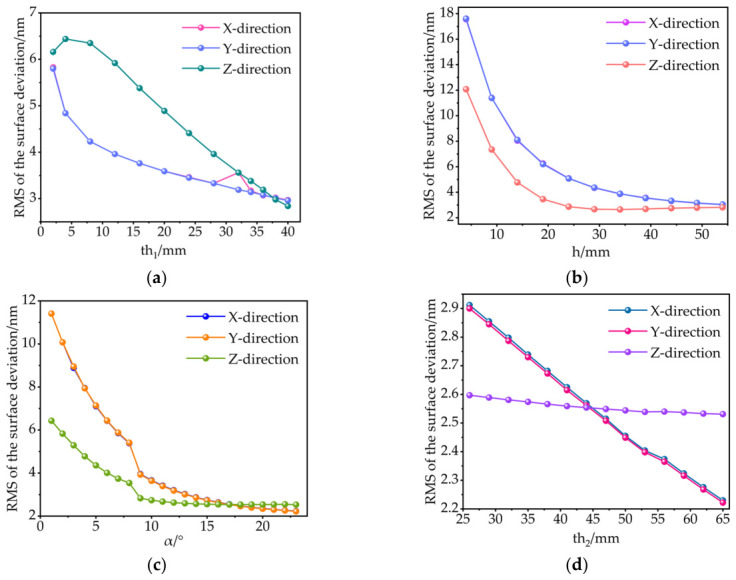
Parameter sensitivity analysis curve: (**a**) The relationship between ${\mathrm{th}}_{1}$ and RMS; (**b**) The relationship between h and RMS; (**c**) The relationship between α and RMS; (**d**) The relationship between ${\mathrm{th}}_{2}$ and RMS.

The thickness ${\mathrm{th}}_{1}$ of the reinforcing rib of the intermediate model is 40 mm, the height h of the reinforcing rib is 59 mm, the end cut angle α is $24^{{}^{\circ}}$, and the thickness ${\mathrm{th}}_{2}$ of the central reinforcing ring is 65 mm. These values are only preliminary estimates based on the results of topology optimization, and there is still room for further optimization. Therefore, the optimization design is carried out again with parameters ${\mathrm{th}}_{1}$, h, α, and ${\mathrm{th}}_{2}$ as variables, and at this time, it is still necessary to pay attention to the key indicator of the accuracy of the mirror surface shape. Figure 5 illustrates the relationship between the RMS of the surface deviation and the aforementioned parameters. As the values of each parameter decrease (i.e., more material is removed), RMS of the surface deviation increases non-linearly. Specifically, within the range of parameter ${\mathrm{th}}_{1}$, the RMS variation peak value of the surface deviation is 3.60 nm; within the range of parameter h, the peak value is 14.57 nm; within the range of parameter α, the peak value is 9.19 nm; and within the range of parameter ${\mathrm{th}}_{2}$, the peak value is 0.68 nm. Based on these data, it is evident that the RMS of the surface deviation is more strongly correlated with the height h of the stiffening rib and the angle α of the end notch, meaning that the RMS of the surface deviation is more sensitive to these two parameters.

**Figure 6:**
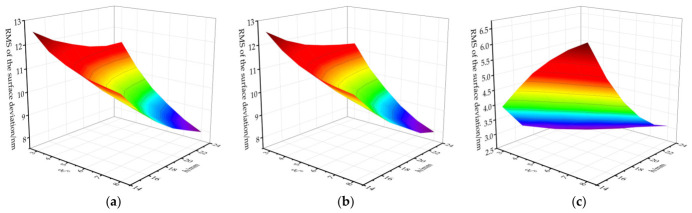
Influence of different axial coupling parameters on surface accuracy: (**a**) X-axis; (**b**) Y-axis; (**c**) Z-axis.

The coupling effect of the two important parameters, h and α, was analyzed, and the results are summarized in Figure 6. Figure 6a–c show the trend of RMS of the surface deviation in the X, Y, and Z axes as a function of the coupling effect of parameters h and α. To a certain extent, it still maintains relatively good monotonicity, but under some extreme values (such as h = 16 mm and $\alpha \;=\;5^{{}^{\circ}}$), the RMS of the surface deviation cannot meet the requirement of being better than 10 nm, which is fatal for large-aperture ultrathin mirrors. Therefore, to ensure that the mirror can provide a good wavefront for the optical system, this paper selects h = 20 mm and $\alpha \;=\;7^{{}^{\circ}}$ as the optimal solutions from a set of numerous feasible solutions. Simultaneously, to ensure the mirror has better impact resistance and mirror surface accuracy retention, this paper preferentially selects ${\mathrm{th}}_{1}\;=\;30$ mm and ${\mathrm{th}}_{2}\;=\;25$ mm. Compared to the model with full material distribution, the final optimized model of the mirror achieves a mass reduction rate of 82.04%.

## 4. Simulation and Analysis

**Figure 7:**
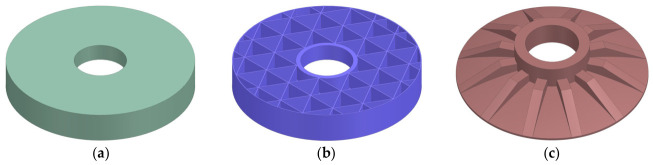
Material distribution of the mirrors: (**a**) Solid; (**b**) Traditional lightweight; (**c**) Optimized (the method proposed in this paper).

The manufacturing and installation of telescopes are carried out under the influence of Earth’s surface temperature and gravitational field. The mechanical-thermal properties of the mirrors under the influence of the Earth’s surface field are crucial. In other words, the good performance of mirrors and their assemblies under terrestrial physical fields is the basis for accurate imaging of space telescopes. To verify the accuracy of the optimization method proposed in this paper, thermal-mechanical and mechanical analyses of the optimized mirror components were performed based on the finite element method. Solid and traditional lightweight mirror components were used as control groups. The material distribution of the three mirrors is shown in Figure 7.

### 4.1. Thermal-Mechanical Analysis

The thermal expansion and deformation of large-aperture ultrathin mirrors caused by temperature changes cannot be ignored. Although telescopes usually have good temperature control systems, the actual working environment temperature still fluctuates, which is one of the starting points for this study. This paper considers the influence of temperature changes on the performance of the mirror under steady-state conditions. The temperature gradient is 0.5 °C, the reference temperature is 20 °C, and the mirror is analyzed within the temperature range of 18.5 °C to 21.5 °C (internal temperature levels of the telescope during on-orbit operation).

**Figure 8:**
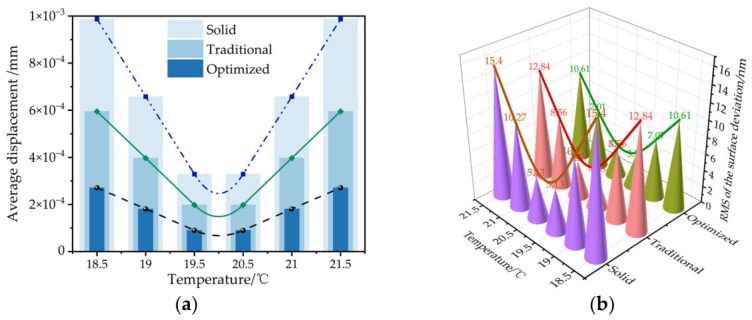
Variation of mirror displacement and the RMS of the surface deviation with temperature: (**a**) Variation of mirror displacement with temperature; (**b**) Variation of the RMS of the surface deviation with temperature.

Table 2 summarizes the mirror displacement values under various temperature conditions. The average displacement of all three types of mirrors reaches its maximum at temperatures of 18.5 °C and 21.5 °C, at $9.87\;\times {\;10}^{-4}$ mm, $5.95\;\times {\;10}^{-4}$ mm, and $2.71\;\times {\;10}^{-4}$ mm, respectively. Compared with the control group, the improvement rates of the average displacement of the optimized mirrors in this paper are 72.54% and 54.45%, respectively. The Akima algorithm was used to interpolate and fit the analysis data, and the fitting curves were plotted in Figure 8a. The displacement of the three types of mirrors showed a basically consistent trend with temperature, and the optimized mirror had the smallest displacement change over the entire temperature range.

To further verify the effect of temperature change on the mirror, the surface accuracy of the above three mirrors was calculated based on Formula (16), and the results are summarized in Table 3. At temperatures of 18.5 °C and 21.5 °C, the RMS of the surface deviation of all three types of mirrors reached their maximum values, at 15.40 nm, 12.84 nm, and 10.61 nm, respectively. The RMS of the surface deviation of the solid and traditional mirrors could no longer meet the accuracy requirement of better than 10 nm. Although the RMS of the surface deviation of the optimized mirror was slightly larger than the requirement, considering sampling error, fitting error, and manufacturing error, it can be considered that the optimized mirror still meets the design requirements. Compared with the control group, the optimized mirror in this paper showed an RMS improvement rate of 31.10% and 17.37%, respectively, with a better mirror shape, reflecting the better temperature adaptability of the optimized mirror.

**Figure 9:**
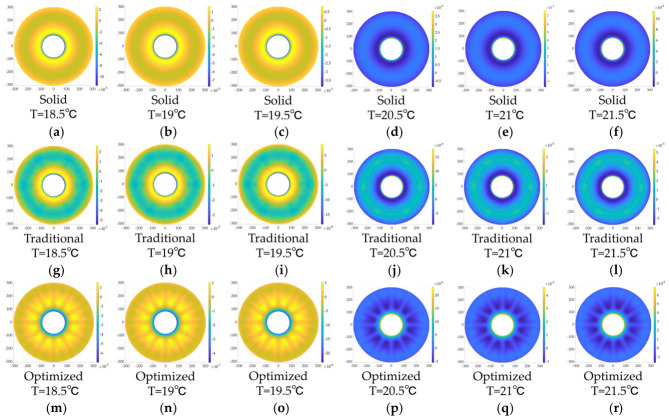
Surface accuracy contour maps at different temperatures: (**a**) 18.5 °C-Solid; (**b**) 19 °C-Solid; (**c**) 19.5 °C-Solid; (**d**) 20.5 °C-Solid; (**e**) 21 °C-Solid; (**f**) 21.5 °C-Solid; (**g**) 18.5 °C-Traditional; (**h**) 19 °C-Traditional; (**i**) 19.5 °C-Traditional; (**j**) 20.5 °C-Traditional; (**k**) 21 °C-Traditional; (**l**) 21.5 °C-Traditional; (**m**) 18.5 °C-Optimized; (**n**) 19 °C-Optimized; (**o**) 19.5 °C-Optimized; (**p**) 20.5 °C-Optimized; (**q**) 21 °C-Optimized; (**r**) 21.5 °C-Optimized.

The RMS value of the surface deviation as a function of temperature is plotted in Figure 8b, which is basically the same as the trend of displacement with temperature, consistent with the basic principles of thermodynamics. Within the temperature range, the optimized mirror shows the smallest change in the RMS peak value, meaning that the effect of temperature load on the optimized mirror is minimal. Figure 9 shows the fitted surface contour plots of the three mirrors at different temperatures.

### 4.2. Mechanical Analysis

**Figure 10:**
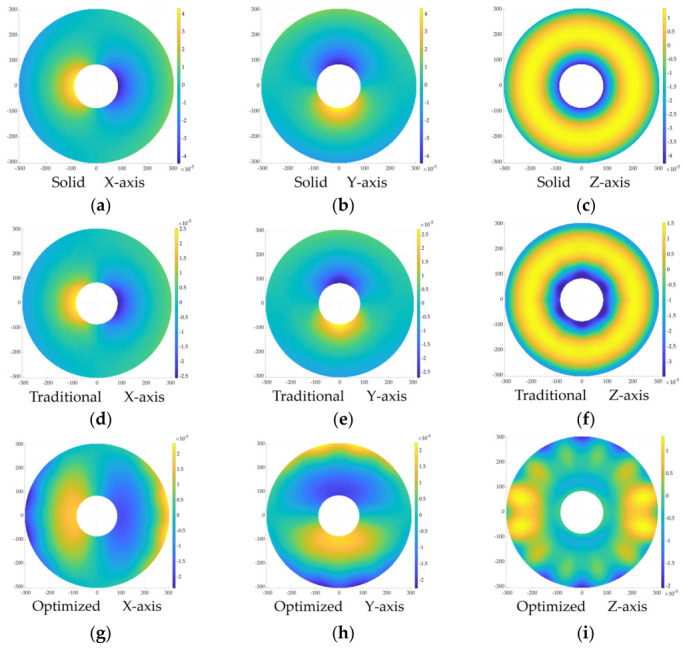
Surface accuracy cloud map of mirrors under the influence of a gravitational physical field: (**a**) X-axis-Solid; (**b**) Y-axis-Solid; (**c**) Z-axis-Solid; (**d**) X-axis-Solid; (**e**) Y-axis-Solid; (**f**) Z-axis-Solid; (**g**) X-axis-Traditional; (**h**) Y-axis-Traditional; (**i**) Z-axis-Traditional.

Excellent mechanical properties are a prerequisite for a mirror to resist various mechanical loads and achieve high-quality imaging. To ensure that a large-aperture ultrathin mirror has good imaging performance under gravity (the gravity here refers to the gravitational acceleration at the internationally defined standard sea level, i.e., $g\;=\;9.81\;m/s^{2}$), a static analysis of the mirror is performed to determine the influence of gravity on the mirror’s wavefront error. Gravity from the X, Y, and Z directions is examined separately, and the RMS of the surface deviation of the mirror surface is used as the evaluation index. Figure 10 shows the surface accuracy contour map of the mirror under the action of multi-directional gravitational physical fields.

Table 4 summarizes the analysis results of the three mirror shapes under the influence of a gravitational field. In the X and Y directions, the surface shape accuracy of both the traditional and optimized mirrors meets the requirements. Although the surface shape accuracy of the solid mirror slightly exceeds the requirements, it still meets the practical needs. In the Z direction, the RMS of the surface deviation of the solid and traditional mirrors are 12.84 nm and 13.35 nm, respectively, both failing to meet the requirements. However, the optimized mirror exhibits excellent surface shape accuracy of 5.94 nm. By comprehensively comparing the RMS of the surface deviation in three directions, it can be found that the optimized mirror has a better ability to maintain the surface shape, which is crucial when installing a telescope.

**Figure 11:**
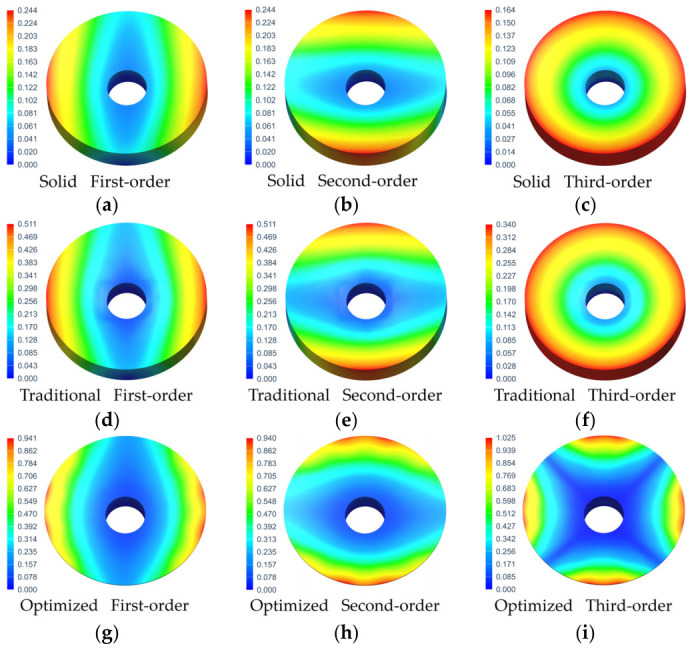
The first three vibration modes of the mirror assembly: (**a**) First-order-Solid; (**b**) Second-order-Solid; (**c**) Third-order-Solid; (**d**) First-order-Solid; (**e**) Second-order-Solid; (**f**) Third-order-Solid; (**g**) First-order-Traditional; (**h**) Second-order-Traditional; (**i**) Third-order-Traditional.

The mirror must possess excellent surface accuracy under static conditions and good mechanical properties under dynamic conditions. This is fundamental to ensuring the telescope can successfully enter space orbit without any irreversible damage. Modal analysis mainly studies the inherent vibration characteristics of the structural system, deriving the system’s natural frequencies and mode shapes. To avoid resonance with other contact components that could cause serious structural damage, the telescope design must effectively separate the system’s natural frequencies from the frequencies of external load excitation. Figure 11 shows the contour plots of the first three modes of the three mirror assemblies mentioned above, and the analysis results are summarized in Table 5. Compared with the control group, the optimized mirror assembly has higher first three modes, namely 1158 Hz, 1164 Hz, and 1238 Hz, which means that the optimized mirror assembly has a lower probability of low-frequency resonance. Because the optimization objective of the method proposed in this paper is to minimize compliance, the mirror can still maintain good stiffness characteristics even after removing a large amount of redundant material.

Besides the influence of static mechanical loads, irregular random vibrations such as rocket engine noise and transportation vibrations also pose a severe challenge to large-aperture ultrathin mirrors. The typical characteristics of random vibration are randomness and non-repeatability. This paper uses power spectral density (PSD) to describe the excitation load of random vibration. Table 6 details the probability distribution of the excitation load and provides the mean square value of the excitation.

**Figure 12:**
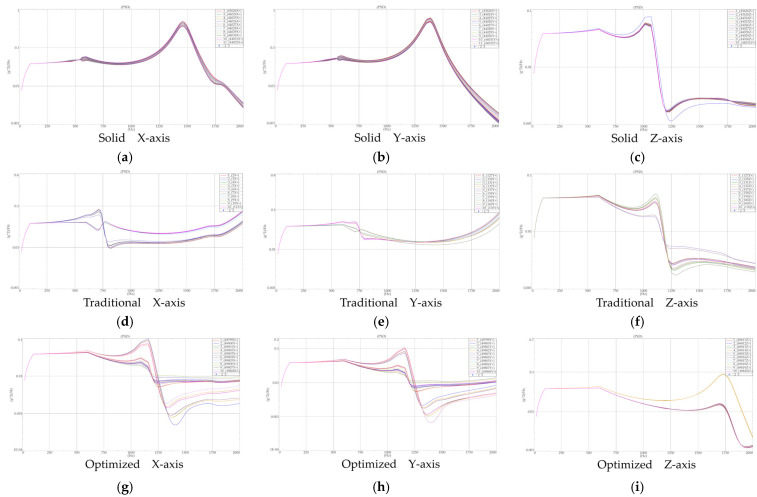
Random vibration response of mirror assemblies with different configurations: (**a**) X-axis-vibration Solid; (**b**) Y-axis-vibration Solid; (**c**) Z-axis-vibration Solid; (**d**) X-axis-vibration Solid; (**e**) Y-axis-vibration Solid; (**f**) Z-axis-vibration Solid; (**g**) X-axis-vibration Traditional; (**h**) Y-axis-vibration Traditional; (**i**) Z-axis-vibration Traditional.

The response curves of the three mirrors in different excitation directions are shown in Figure 12. In order to improve the reliability of the results, the number of sampled feature points in this paper is no less than 15,000, and all feature points of key parts such as mirror surface, contact surface, and stress concentration point need to be selected.

Table 7 summarizes the response results of the mirror assembly under random vibration. The power spectral density amplification factors of the optimized mirror are 3.09, 3.27, and 4.39 in the three directions, respectively. Compared with the control group, only the vibration response results of the optimized mirror can simultaneously meet the requirement of response amplification factor less than 4.5 in all three directions. Moreover, the power spectral density amplification factors of the solid mirror assembly in the X and Y directions far exceed the requirements, which is destructive to the structure. This demonstrates that it is necessary and beneficial to reasonably remove redundant materials.

## 5. Discussion

To achieve excellent thermodynamic performance in large-aperture ultrathin mirrors, this paper proposes a mirror optimization method based on multiphysics coupling. The interaction between thermal and mechanical physical fields is established, and the problems of non-smoothness of the max(·) function and the inability to solve for sensitivity are solved by using the P-norm approximation method. Combined with parameter optimization methods, the final optimization model of the mirror is determined, and a surface shape accuracy fitting method is established to evaluate the deviation of the mirror surface shape. The proposed method was validated using a 610 mm diameter mirror as the research object. Compared with solid mirrors and traditional lightweight mirrors, the mirror optimized using the proposed method shows significant improvements in mass reduction ratio and thermo-mechanical properties, which aligns with the basic optimization theory presented in this paper. The proposed optimization method couples the thermal load with the gravitational load in the form of an equivalent load, incorporating the coupled load into the mechanical equilibrium equations of topology optimization. Furthermore, a steady-state heat conduction finite element equilibrium equation is introduced to describe the balance between heat conduction and the thermal load. Minimizing compliance is the optimization objective, ensuring that the mirror possesses good stiffness characteristics after removing redundant materials.

This paper provides a new approach to the lightweight design of large-aperture ultrathin mirrors and achieves synergistic optimization of mirrors that simultaneously consider both lightweight efficiency and thermodynamic properties.

While the optimization method proposed in this paper has many advantages, it also has some shortcomings:

1. In this study, the maximum displacement of the mirror node is used as a constraint, and the optical performance of the mirror is not directly considered.
2. This paper focuses on the mirror and its components, without considering the actual propagation process of light.
3. This paper takes minimizing compliance as the optimization objective, but in practical applications, it may be necessary to simultaneously optimize multiple performance targets, lacking a multi-objective optimization process.

Future research should focus directly on the optical performance of the telescope system, fully consider the multi-objective requirements in the mirror optimization process, and explore efficient methods to find the optimal solution to multi-objective problems. In addition, the optimization methods and analysis results in this paper are all based on numerical simulation models, which inevitably differ from actual manufacturing. This section discusses in detail the uncertainties and model limitations in the research:

1. Sensitivity of structure to manufacturing tolerances and assembly errors: In the topology optimization process, this paper initially takes into account both structural regularity and manufacturability by using structured meshes, dimensional constraints, and boundary constraints. Based on the optimization results, further design and parameter optimization are carried out to reduce the sensitivity to manufacturing tolerances. However, no specific optimization for the accumulation of manufacturing tolerances and assembly errors is carried out. In subsequent research, Monte Carlo uncertainty analysis can be introduced to quantify the numerical relationship between tolerances and errors and structural performance.
2. Material performance uncertainty: Factors such as process fluctuations and residual stress can cause discrepancies between the actual material’s elastic modulus, coefficient of thermal expansion, and other key parameters and their ideal values. In this study, all material performance parameters were selected as intermediate values, and measured data published by multiple manufacturers were compared. To further reduce the impact of material performance uncertainty, future research could analyze extreme parameter values and conduct experimental tests on the purchased raw materials before establishing a simulation model.
3. Impact of installation method: This study seriously considered actual installation methods during modeling, constraining the model through adhesive contact, fixing constraints, and temperature boundaries to maintain consistency with actual assembly conditions. Future research could focus on refined modeling of contact interfaces and diverse designs based on optimization results to improve mirror accuracy.
4. Limitations of the temperature model: This study focuses on the impact of changes in the overall temperature level of the mirror on the mirror’s accuracy, neglecting the effects of instantaneous thermal loads such as light radiation and heat conduction from electronic components. Future research can improve the optimization method under transient conditions.

## 6. Conclusions

Focusing on the key technologies of large-aperture ultrathin mirrors while ensuring good thermodynamic performance, this paper proposes a mirror optimization method based on multiphysics coupling. Based on the finite element method and thermoelastic theory, the interaction relationship between the temperature and force physical fields is established. To determine the optimal material distribution of the mirror, a topology optimization equation is established using equivalent loads. The problem of non-smoothness of the max(·) function and the inability to solve for sensitivity is solved by using the P-norm to approximate the max(·) function. Combined with parameter optimization methods, the final optimized model of the mirror is determined, and a surface shape accuracy fitting method is established to evaluate the deviation of the mirror surface shape.

To verify the accuracy of the proposed method, this paper uses a 610 mm diameter mirror as the research object. Combining topology optimization and parameter optimization methods, the optimized mirror’s structural layout was determined. Compared to a solid mirror, the optimized mirror achieves a mass reduction rate of 82.04%. Using solid and traditional lightweight mirrors as control groups, thermo-mechanical analysis was performed. The optimized mirror showed superior surface accuracy across the entire temperature range compared to the control group, and still met the 10 nm requirement at boundary temperatures. The RMS of the surface deviation of the optimized mirror under the action of the gravitational physical field are 9.07 nm, 9.07 nm and 5.94 nm, respectively, which meet the requirements. In terms of dynamic response, the optimized mirror assembly showed significant improvements in the first three modes, and also exhibited good performance in random vibration responses in all three directions. The optimized mirror had the highest response amplification factor in the Z direction, at 4.39, below the threshold of 4.5 required to maintain the structural stability. The above analysis results demonstrate that the mirror optimization method based on multi-physics coupling proposed in this paper is correct and feasible. It not only improves the mass reduction rate of the mirror, but also improves the environmental adaptability to a certain extent.

## Figures and Tables

**Table 1:** Key parameters of the mirror.

Item	Parameter
Diameter/mm	610.00
Material	SiC
Radius of curvature/mm	1009.30
Mass/kg	64.16
Mirror surface thickness/mm	5.00
Mirror body thickness/mm	54.00

**Table 2:** Comparison of different forms of mirror displacement at different temperatures.

Temperature/°C	Maximum Displacement Value/$10^{-4}$ mm			Average Displacement Value/$10^{-4}$ mm		
	Solid	Traditional	Optimized	Solid	Traditional	Optimized
18.5	15.20	9.31	4.76	9.87	5.95	2.71
19	10.12	6.21	3.17	6.58	3.97	1.81
19.5	5.06	3.11	1.59	3.29	1.98	0.90
20.5	5.06	3.11	1.59	3.29	1.98	0.90
21	10.12	6.21	3.17	6.58	3.97	1.81
21.5	15.20	9.31	4.76	9.87	5.95	2.71

**Table 3:** Comparison of the surface accuracy of different types of mirror surfaces at different temperatures.

Temperature/°C	RMS of the Surface Deviation/nm		
	Solid	Traditional	Optimized
18.5	15.40	12.84	10.61
19	10.27	8.56	7.07
19.5	5.13	4.28	3.54
20.5	5.13	4.28	3.54
21	10.27	8.56	7.01
21.5	15.40	12.84	10.61

**Table 4:** Comparison of surface accuracy of mirrors under the influence of gravitational physical field.

Direction	RMS of the Surface Deviation/nm		
	Solid	Traditional	Optimized
X-axis	10.92	6.13	9.07
Y-axis	10.81	6.35	9.07
Z-axis	12.84	13.35	5.94

**Table 5:** First three natural frequencies of the mirror assembly.

Structural Form	First-Order Mode/Hz	Second-Order Mode/Hz	Third-Order Mode/Hz
Solid	545	551	603
Traditional	740	743	1062
Optimized	1158	1164	1238

**Table 6:** Excitation conditions for random vibration.

Frequency/Hz	Power Spectral Density	Total Root Mean Square Acceleration/grms
20~100	+3 dB/oct	6.07 g
100~600	0.038 $g^{2}/\mathrm{Hz}$	
600~2000	−6 dB/oct	

**Table 7:** Response results of random vibration.

Direction	Structural Form	Power Spectral Density/$g^{2}/\mathrm{Hz}$	Frequency Point/Hz	Response Amplification Factor
X-axis	Solid	0.469	1462	8.56
	Traditional	0.082	712	1.74
	Optimized	0.098	1157	3.09
Y-axis	Solid	0.590	1386	9.08
	Traditional	0.088	2000	5.05
	Optimized	0.110	1157	3.27
Z-axis	Solid	0.075	1065	2.49
	Traditional	0.045	1109	2.01
	Optimized	0.089	1730	4.39

## Data Availability

The original contributions presented in this study are included in the article. Further inquiries can be directed to the corresponding author.
